# Accuracy of genomic prediction of maternal traits in pigs using Bayesian variable selection methods

**DOI:** 10.1111/jbg.12729

**Published:** 2022-06-27

**Authors:** Maria V. Kjetså, Arne B. Gjuvsland, Øyvind Nordbø, Eli Grindflek, Theo Meuwissen

**Affiliations:** ^1^ Norwegian University of Life Sciences, Faculty of Biosciences Ås Norway; ^2^ Norsvin SA Hamar Norway

**Keywords:** Bayesian genomic prediction, genomic prediction accuracy, genomic selection, maternal traits

## Abstract

The aim of this study was to compare three methods of genomic prediction: GBLUP, BayesC and BayesGC for genomic prediction of six maternal traits in Landrace sows using a panel of 660 K SNPs. The effects of different priors for the Bayesian methods were also investigated. GBLUP does not take the genetic architecture into account as all SNPs are assumed to have equally sized effects and relies heavily on the relationships between the animals for accurate predictions. Bayesian approaches rely on both fitting SNPs that describe relationships between animals in addition to fitting single SNP effects directly. Both the relationship between the animals and single SNP effects are important for accurate predictions. Maternal traits in sows are often more difficult to record and have lower heritabilities. BayesGC was generally the method with the higher accuracy, although its accuracy was for some traits matched by that of GBLUP and for others by that of BayesC. For piglet mortality within 3 weeks, BayesGC achieved up to 9.2% higher accuracy. For many of the traits, however, the methods did not show significant differences in accuracies.

## 1 INTRODUCTION

Genomic prediction (GP) (Meuwissen et al., 2001) is a method to predict breeding values (GEBVs) in animal and plant breeding. GP predicts the GEBVs by using a reference population of animals with both phenotype and marker information to estimate marker effects. Meuwissen et al. proposed three methods for genomic prediction: two Bayesian variable selection methods (BayesA and B) and a linear marker effects model estimating marker effects from single‐nucleotide polymorphisms (SNPs) using best linear unbiased prediction (BLUP), referred to as SNP‐BLUP. An alternative method of SNP‐BLUP is to use a marker‐derived genomic relationship matrix (often called a **G**‐matrix) as a covariance matrix when solving mixed‐model equations (MME) (VanRaden, 2008) referred to as genomic best linear unbiased predictions (GBLUP). The two methods, SNP‐BLUP and GBLUP, are mathematically equivalent (Strandén & Garrick, 2009; VanRaden, 2008).

All markers are assumed to have equal weight in the prediction for the linear models, while Bayesian methods try to differentiate SNPs relative to their importance. Markers associated with causal mutations get a higher relative weight, and markers not linked to causal loci are down‐weighted, thus only giving weights to the most important SNPs (Meuwissen et al., 2001; Verbyla et al., 2010). Several alternative Bayesian variable selection methods are proposed, often referred to as the “Bayesian Alphabet” (Gianola et al., 2009). Differences between the methods are the prior distributions used for the estimation of SNP effects. For example, BayesA uses one t‐distribution for SNP effects, while BayesB has a mixture of a t‐distribution with probability π, and a null effect with probability 1‐π. BayesC (Habier et al., 2011) is similar to BayesB, as both have a mixture distribution prior, where one has a null effect. However, BayesC uses a normal distribution instead of a t‐distribution and assumes a common variance for all SNPs, while BayesB assumes SNP‐specific variances. BayesR uses four normal distributions, where one of them has a null effect (Erbe et al., 2012). The recently proposed BayesGC method (Meuwissen et al., 2021), fits a polygenic effect through a **G**‐matrix in addition to a BayesC term. Hence, BayesGC fits many SNPs with a small effect through the **G**‐matrix and a group of SNPs selected by the model with more significant effects through the BayesC term.

In this paper, we look at the genomic prediction of maternal traits in landrace pigs, which are considered complex traits with a low to moderate heritability and explore the effect of the genetic architecture on the prediction accuracy. Specifically, we look at the traits; the total number of born piglets (**TNB**), number of stillborn piglets (**STB**), piglet mortality within 3 weeks, i.e. number of piglets dead after birth and until 3 weeks (**M3W**), total litter weight at 3 weeks (**LW3W**), sow shoulder lesions (**SHL**) and the sow's body condition score (**BCS**). These maternal traits were included in the breeding goal for Topigs Norsvin at the time of recording (Eriksen, 2018).

Maternal traits in pigs are related to the sow's ability to produce and raise offspring. Maternal traits are essential for efficiency in pig production, the economy and animal welfare (Ocepek & Andersen, 2017). An ideal sow produces a litter of piglets corresponding to the number of functional teats available, and all the piglets born survive until weaning. Furthermore, the piglets should grow evenly, and the sow should not spend all her resources on the litter, implying that she maintains a good body condition score and does not develop shoulder lesions.

Simulation studies have shown great potential for using genomic prediction methods to predict maternal traits in pigs (Lillehammer et al., 2011, 2013). Although few studies have reported genomic prediction accuracies for maternal traits in pigs (Tan et al., 2017), there are very few that have reported prediction accuracies for Bayesian genomic prediction methods for maternal traits. Some have looked at Bayesian methods in growth and reproduction traits (Song et al., 2017) and slaughter traits (Salek Ardestani et al., 2021). Although the basis of inheritance and breeding is the same across livestock species, their differences in breeding structure, genetic architecture and trait biology make it important to study the different prediction methods across the species (Samorè & Fontanesi, 2016).

This study aimed to determine the prediction accuracy of six maternal traits in Landrace sows using a panel of 660 k SNP markers and a large reference population (9–15 thousand reference animals). The study also compares three methods of genomic prediction: GBLUP (VanRaden, 2008), BayesC (Habier et al., 2011) and BayesGC (Meuwissen et al., 2021).

## 2 MATERIALS AND METHODS

### 2.1 Phenotypic data

The phenotypic data consisted of records from 15,703 unique individual Landrace sows with at least one record for one of the six traits; the total number of born piglets (**TNB**), number of stillborn piglets (**STB**), piglet mortality within 3 weeks, i.e. number of piglets dead after birth and until 3 weeks (**M3W**), total litter weight at 3 weeks (**LW3W**), sow shoulder lesions (**SHL**) and the sow's body condition score (**BCS**). Of the 15,703 sows, 10,306 had records for all six traits. The traits were recorded between 2008 and 2019. Each of the different traits had between 10,611–15,690 phenotypic records (see Table 1).

**TABLE 1:** Number (*n*) of animals with records for each trait and partition into the reference and validation population, and mean (*m*) number of parity records in each trait

Trait^a^	Total, *n*	Reference, *n*	Validation, *n*	Parity, *m*
TNB	15,690	14,513	1177	2.5
STB	15,690	14,513	1177	2.5
M3W	10,611	9466	1145	1.7
LW3W	10,804	9656	1148	1.7
SHL	15,084	13,934	1150	2.2
BCS	15,084	13,933	1151	2.2

^a^ Total number born (TNB), number of stillborn piglets (STB), piglet mortality within 3 weeks, i.e. number of piglets dead after birth and until 3 weeks (M3W), total litter weight at 3 weeks (LW3W), sow shoulder lesions (SHL) and the sow's body condition score (BCS).

Yield Deviations (VanRaden & Wiggans, 1991) for the six traits were derived from the commercial breeding value evaluations from Topigs Norsvin. There were multiple records for each trait, as we had one YD for each parity. The maximum number of parities recorded for each trait was 6, and the mean number of parities recorded for each trait is shown in Table 1. Because the software used for the Bayesian variable selection models (Meuwissen et al., 2021) could not handle multiple records per animal, we used the average YD for each sow, with a weighting of each record corresponding to the effective number of records calculated by the formula $\frac{n\cdot \left(1+\lambda \right)\;}{\left(n+\lambda \right)}$ where λ is $\sigma_{e}^{2}/\sigma_{\mathrm{pe}}^{2}$ and *n* is the number of records for each individual, $\sigma_{e}^{2}$ is the residual variance and $\sigma_{\mathrm{pe}}^{2}$ is the permanent environmental variance (λ was obtained from Topigs Norsvin's breeding value evaluation).

### 2.2 Genotype data

The sows were genotyped with varying SNP densities and imputed to a 660 K‐genotype density. Of the 15,703 sows, 526 were genotyped on a 10 K chip (GGP Porcine LD), and the rest were genotyped on medium density chips: Illumina PorcineSNP60 (60 K) and two Illumina GeneSeek custom chips (80 K and 50 K). All genotypes were imputed using Fimpute v2.2 (Sargolzaei et al., 2014), first to the 50 K chip, and then to the 660 K Axiom Porcine Genotyping Array with reference genotypes from 467 Landrace animals. After quality control, the 660 K High‐Density genotype data had a total of 429,403 SNPs with MAF >0.01.

### 2.3 Validation and reference data

The ~1000 youngest sows were used for validation of the predictions, in order to imitate a typical genomic breeding program where one wishes to predict the breeding values of young animals before they have their own recorded traits. This was done by masking their phenotypic records in the analysis and using them for validation. The number of validation sows was between 1145 and 1177 (Table 1). The rest of the animals was used as the reference data with both phenotypic and genotype records. Our smallest reference dataset consisted of 9466 animals for the trait M3W and the largest of 14,513 animals for traits TNB and STB (Table 1).

### 2.4 Prediction accuracy and regression coefficients

The accuracy of prediction for all methods was estimated as:

$$r_{\text{pred}}=\frac{\mathrm{cor}\left(\text{GEBV},\mathrm{YD}\right)}{\sqrt{h^{2}}},$$

and the bias (coefficient of regression) was the calculated slope (*b*) of the linear regression Y = *a* + *b*X, where a is the intercept, Y is the yield deviation (YD) and X is the genomic estimated breeding value (GEBV) of the sows in the validation datasets, estimated with only marker information and not phenotypic records. *h* ^2^ is the heritability of the trait and was estimated on the full dataset.

### 2.5 Variance components and GBLUP

We estimated variance components for each trait using the pedigree relationship matrix and the DMUAI package from the DMU software (Madsen & Jensen, 2013). The variance components were estimated on the full dataset (i.e. both the reference and validation animals). The model for the variance component estimations was as follows:

y=1μ+Zu+e,

where ***y*** is a vector of the average YD of a sow, **1** is a vector of ones corresponding to the size of ***y***, *μ* is the mean, ***Z*** is a design matrix linking individuals to the phenotype, ***u*** is the random effect of the individual animal (***u*** ~ N(0, **A** *σ*_*u*_^2^), where **A** is the pedigree relationship matrix and ***e*** is the residual effect (***e*** ~ N[0, **D** *σ*_*e*_^2^]), where **D** is a diagonal matrix where the diagonals are the inverses of the effective number of records. The same model was used for the GBLUP analyses except that the individual animal effect was modelled as (***u*** ~ N[0, **G** *σ*_*u*_^2^]). The variance components used were from the above pedigree‐based estimates. The **G**‐matrix was calculated using the VanRaden method 1 (VanRaden, 2007).

### 2.6 BayesC

The model for BayesC (Habier et al., 2011) was:

$$y=1\;\mu +\sum_{i}I_{i}x_{i}s_{i}+e.$$

where ***y*** is a vector of Yield Deviations, **1** is a vector of ones, *μ* is the overall mean, $x_{i}$ is a vector of genotypes for SNP *i* containing ‐2*p*_*i*_ for homozygote individuals, 1‐2*p*_*i*_ for heterozygotes and 2‐2*p*_*i*_ for the alternative homozygote genotype with *p*_*i*_ being the allele frequency of SNP *i*, and $I_{i}$ is an indicator of whether the SNP *i* is in the model in a particular MCMC cycle or not (0/1), where the prior probability of $I_{i}$ being equal to 1 is denoted by *π* (values in Table 2), $s_{i}$ is the SNP effect, where if the SNP *i* is in the model: $s_{i}$ ~ N(0, $\sigma_{m}^{2}$), ***e*** is the residual with variance ***e*** ~ N(0 **D** *σ*_*e*_^2^), where **D** is a diagonal matrix where the diagonals are the inverses of the effective number of records and *σ*_*e*_^2^ is the residual variance estimated from the variance component estimations (Table 5). The Markov Chain Monte Carlo (MCMC) chain was run for 20,000 Gibbs cycles using 4000 burn‐in cycles, in two distinct chains.

**TABLE 2:** *π* values used for BayesC and BayesGC methods at different fractions of total genetic variance explained by a single‐fitted SNP (Fr)

Fr	BayesGC_10^a^	BayesGC_50^b^	BayesGC_90^c^	BayesC
1/100	0.00002	0.00012	0.00021	0.0002
1/500	0.00012	0.00058	0.00105	0.0012
1/1000	0.00023	0.00116	0.00210	0.0023
1/5000	0.00116	0.00582	0.01048	0.0116
1/10,000	0.00233	0.01164	0.02096	0.0233

^a^ BayesGC_10 is Bayes_GC with 10% marker variance and 90% polygenic variance.

^b^ BayesGC_50 has 50% marker variance and 50% polygenic variance.

^c^ BayesGC_90 has 90% marker variance and 10% polygenic variance.

We used the same variance components as for the GBLUP analyses; however, the total genetic variance $\sigma_{u}^{2}$ was partitioned. In the following, we describe how the total genetic variance $\sigma_{u}^{2}$ (see Table 4) is partitioned over the fitted SNPs for the Bayes C method:

$$\sigma_{m}^{2}=\frac{\mathrm{Fr}\cdot \sigma_{u}^{2}}{\bar{\mathrm{HET}}},$$

where $\sigma_{m}^{2}$ is the genetic variance explained by a single SNP,

Fr is the fraction of the total genetic variance explained by a single fitted SNP, i.e. 1/1000 when we assume each SNP explains 1/1000th of the genetic variance. We test different values of Fr, namely 1/100, 1/500, 1/1000, 1/5000 and 1/10,000, respectively.

$$\bar{\mathrm{HET}}=\text{average heterozygosity}=\frac{2\sum p_{i}\;\left(1-p_{i}\right)}{N_{\text{loci}}},$$

where *p*_*i*_ is the allele frequency of locus *i* and $N_{\text{loci}}$ is the total number of loci.

For a Bayes C model, this would mean using a prior probability of fitting an SNP of:

$$\pi_{c}=\frac{1/\mathrm{Fr}}{N_{\text{loci}}}.$$

Such that the total genetic variance is $\sigma_{u}^{2}=\pi_{c}\cdot N_{\text{loci}}\cdot \bar{\mathrm{HET}}\cdot \sigma_{m}^{2}$.

### 2.7 BayesGC

The BayesGC model is as follows:

$$y=1\;\mu +\mathrm{Zu}+\sum_{i}I_{i}x_{i}s_{i}+e,$$

where ***y*** is a vector of the Yield Deviations, **1** is a vector of ones, μ is the overall mean, ***Z*** is a design matrix that links individuals to the ***y***, ***u*** is a vector of random polygenic effects with variance *V*(***u***) = **G** $\sigma_{\mathrm{pol}}^{2}$, $x_{i}$ is the vector of genotypes for SNP *i* coded as for BayesC. $I_{i}$ is an indicator of whether SNP *i* is in the model in an MCMC cycle or not (0/1) and the prior probability of $I_{i}$ being equal to 1 is π (listed in Table 2), $s_{i}$ is the SNP effect, where if the SNP *i* is in the model: $s_{i}$ ~ N(0, $\sigma_{m}^{2}$), ***e*** is the residual with variance ***e*** ~ N(0, **D** *σ*_*e*_^2^) where **D** is a diagonal matrix where the diagonals are the inverses of the effective number of records and *σ*_*e*_^2^ is the residual variance estimated from the variance component estimations (Table 5). The MCMC chain was run for 4000 burn‐in cycles and a total of 20,000 Gibbs cycles for two independent chains. The EBVs from the two Gibbs chains for both BayesC and BayesGC had a correlation of >0.9999, and thus, the EBVs were assumed to be converged, and the results presented for both BayesC and BayesGC are the average of two Gibbs chains.

The BayesGC model basically fits the previous two models (GBLUP and BayesC) simultaneously, i.e. it fits a polygenic and a BayesC term. The polygenic effect is fitted using the genomic relationship matrix (**G**) as in the GBLUP model. The BayesC term assumes SNPs to have normally distributed effects with probability (*π*) or an effect of 0 with probability (1 − *π*).

In the following, we describe how the total genetic variance $\sigma_{u}^{2}$ is partitioned over the fitted SNPs and the polygenic effect, following the method described in Kjetså et al. (2020). For BayesGC, we need an assumption on the fraction of the variance that is explained by the individually fitted SNPs in the BayesC term of the model. In addition, the total genetic variance $\sigma_{u}^{2}\;$should not be affected by the partitioning of the variance across the SNPs and the polygenic effect. Let q be the fraction of $\sigma_{u}^{2}$ explained by the BayesC term, then the variance explained by the polygenic effect is $\sigma_{\mathrm{pol}}^{2}$ = (1 − *q*) $\sigma_{u}^{2}$. Hence,

$$\sigma_{u}^{2}=\sigma_{\mathrm{pol}}^{2}+q\cdot \pi \cdot \text{loci}\cdot \bar{\mathrm{HET}}\cdot \sigma_{m}^{2},$$

It follows that

$$\pi_{\mathrm{gc}}=q\cdot \pi_{c},$$

where $\pi_{\mathrm{gc}}$ is the π value used for the BayesGC method. Four different values of *q* were tested for BayesGC, *q* = 0.1, 0.5 and 0.9 corresponding to the BayesC term with fitted marker effects explaining 10%, 50% or 90% of the total genetic variance (denoted BayesGC_10, BayesGC_50, BayesGC_90, respectively), with the rest of the variance 1 − *q* explained by the polygenic effect through the **G**‐matrix. The values of $\sigma_{m}^{2}$ used are shown in Table 3 and the values of $\sigma_{\mathrm{pol}}^{2}$ are shown in Table 4.

**TABLE 3:** Priors of variance of a single marker ($\sigma_{m}^{2}$) used in the BayesC and BayesGC methods under the different priors for the fraction of total genetic variance explained by a single‐fitted SNP (Fr) where $\sigma_{m}^{2}$ = $\frac{\mathrm{Fr}\cdot \sigma_{u}^{2}}{\bar{\mathrm{HET}}}$ for each trait

Fr	$\sigma_{m}^{2}$					
	TNB^a^	STB^a^	M3W^a^	LW3W^a^	SHL^a^	BCS^a^
1/100	0.03196	0.00509	0.00518	0.53802	0.00259	0.00274
1/500	0.00639	0.00102	0.00104	0.10760	0.00052	0.00055
1/1000	0.00320	0.00051	0.00052	0.05380	0.00026	0.00027
1/5000	0.00064	0.00010	0.00010	0.01076	0.00005	0.00005
1/10,000	0.00032	0.00005	0.00005	0.00538	0.00003	0.00003

^a^ Total number born (TNB), number of stillborn piglets (STB), piglet mortality within 3 weeks, i.e. number of piglets dead after birth and until 3 weeks (M3W), total litter weight at 3 weeks (LW3W), sow shoulder lesions (SHL) and the sow's body condition score (BCS).

**TABLE 4:** Priors for variance attributed to the polygenic effect for the different traits for the different BayesGC methods

	Trait	BayesGC_10^a^	BayesGC_50^b^	BayesGC_90^c^
$\sigma_{\mathrm{pol}}^{2}$	TNB	0.944	0.525	0.105
	STB	0.150	0.084	0.017
	M3W	0.153	0.085	0.017
	LW3W	15.89	8.830	1.766
	SHL	0.077	0.043	0.009
	BCS	0.081	0.045	0.009

^a^ BayesGC_10 is BayesGC with 10% marker variance and 90% polygenic variance.

^b^ BayesGC_50 has 50% marker variance and 50% polygenic variance.

^c^ BayesGC_90 has 90% marker variance and 10% polygenic variance.

## 3 RESULTS

**FIGURE 1:**
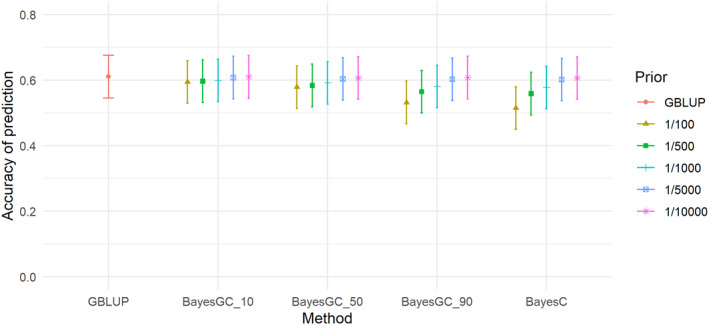
The accuracy of prediction for the trait total number born (TNB), from the different prediction methods at the different priors for fraction of variance explained by a single SNP (bars denote standard errors) [Colour figure can be viewed at wileyonlinelibrary.com]

**FIGURE 2:**
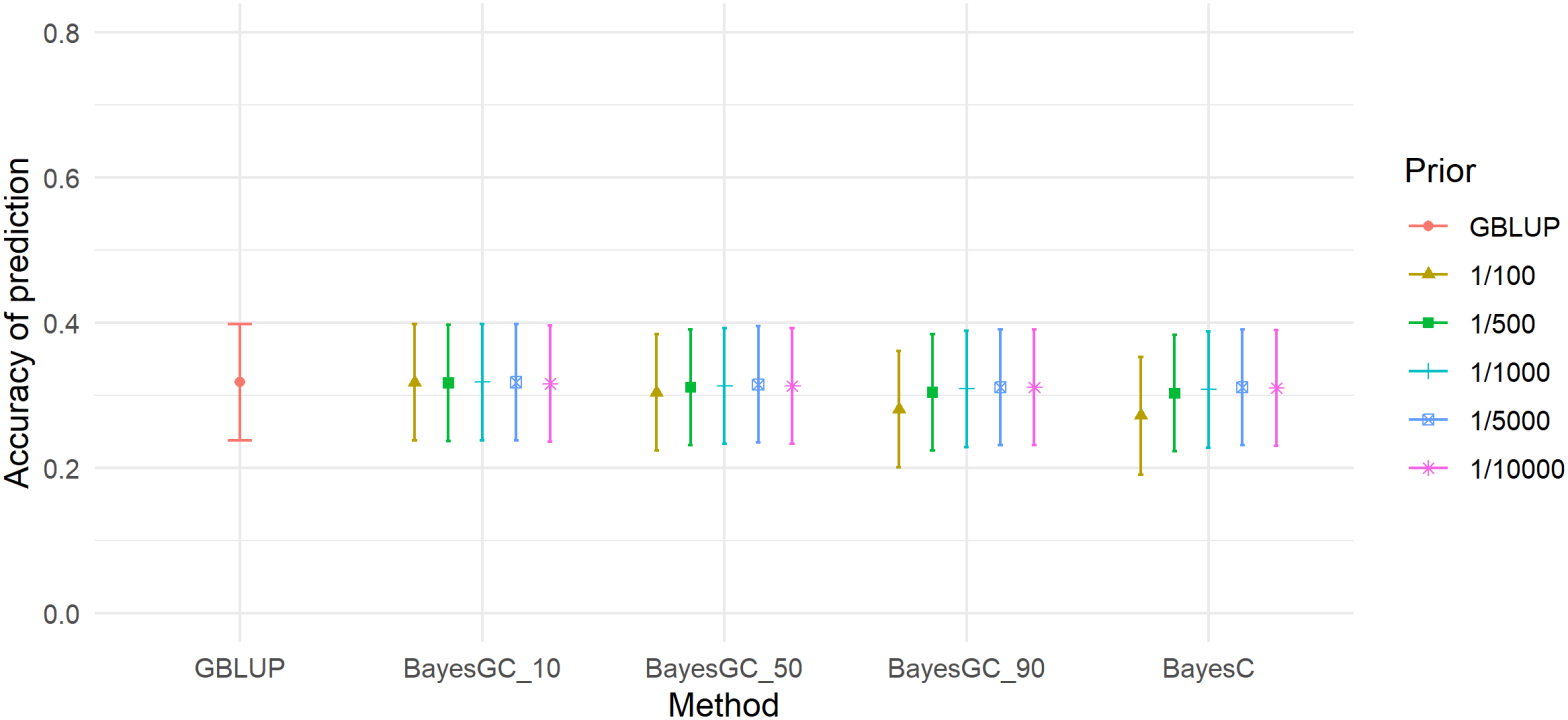
The accuracy of prediction for the trait number of stillborn (STB), from the different prediction methods at the different priors for fraction of variance explained by a single SNP (bars denote standard errors) [Colour figure can be viewed at wileyonlinelibrary.com]

The heritabilities of the traits ranged from 0.09 (M3W) to 0.34 (SHL) (Table 5). M3W had the lowest heritability of 0.09, followed by STB and TNB with moderate heritabilities of 0.13 and 0.19. LW3W, BCS and SHL had the highest heritabilities with 0.31, 0.31 and 0.34, respectively (Table 5). For the trait total number born (TNB), the highest accuracy was achieved at 0.610–0.611 for GBLUP and BayesGC_10 (Table 6) and the method giving the lowest prediction accuracy is BayesC (Fr 1/100) (Figure 1), which achieved an accuracy of 0.515 for TNB. For all the Bayesian methods (BayesGC_10, BayesGC_50, BayesGC_90 and BayesC), fitting more SNPs (Fr = 1/10000) gave the highest accuracy of prediction for the trait TNB. The accuracy of prediction for Stillborn (STB; Figure 2), is lower than the other traits.

**TABLE 5:** The estimated total genetic variance ($\sigma_{u}^{2}$), residual variance ($\sigma_{e}^{2}$) and heritabilities ($h^{2}$) for the six maternal traits

Trait^a^	$\sigma_{u}^{2}$	$\sigma_{e}^{2}$	$h^{2}$
TNB	1.049	4.490	0.189
STB	0.167	1.125	0.130
M3W	0.170	1.791	0.087
LW3W	17.66	39.99	0.306
SHL	0.085	0.163	0.343
BCS	0.090	0.203	0.307

^a^ total number born (TNB), number of stillborn piglets (STB), piglet mortality within 3 weeks, i.e. number of piglets dead after birth and until 3 weeks (M3W), total litter weight at 3 weeks (LW3W), sow shoulder lesions (SHL) and the sow's body condition score (BCS).

**TABLE 6:** Accuracy, standard error (*SE*) and regression coefficients (*b*) for each trait from the method and fraction of the total genetic variance explained by a single‐fitted SNP (Fr) yielding the highest accuracy for each trait

Trait^a^	Method	Fr	Accuracy	*SE*	*b*
TNB	BayesGC_10^b^	1/10,000	0.610	0.07	0.97
	BayesC	1/10,000	0.607	0.07	0.95
	GBLUP	—	0.611	0.07	0.98
STB	BayesGC_10^b^	1/5000	0.318	0.08	0.50
	BayesC	1/5000	0.311	0.08	0.49
	GBLUP	—	0.318	0.08	0.50
M3W	BayesGC_50^c^	1/100	0.484	0.10	0.79
	BayesC	1/500	0.464	0.10	0.74
	GBLUP	—	0.441	0.10	0.74
LW3W	BayesGC_90^d^	1/10,000	0.722	0.05	1.17
	BayesC	1/10,000	0.718	0.05	1.04
	GBLUP	—	0.717	0.05	1.04
SHL	BayesGC_50^c^	1/100	0.418	0.05	0.44
	BayesC	1/5000	0.409	0.05	0.41
	GBLUP	—	0.406	0.05	0.41
BCS	BayesGC_50^c^	1/5000	0.518	0.05	0.70
	BayesC	1/5000	0.518	0.05	0.70
	GBLUP	—	0.511	0.05	0.70

^a^ Total number born (TNB), number of stillborn piglets (STB), piglet mortality within 3 weeks, i.e. number of piglets dead after birth and until 3 weeks (M3W), total litter weight at 3 weeks (LW3W), sow shoulder lesions (SHL) and the sow's body condition score (BCS).

^b^ BayesGC_10 is BayesGC with 10% marker variance and 90% polygenic variance.

^c^ BayesGC_50 has 50% marker variance and 50% polygenic variance.

^d^ BayesGC_90 has 90% marker variance and 10% polygenic variance.

**FIGURE 3:**
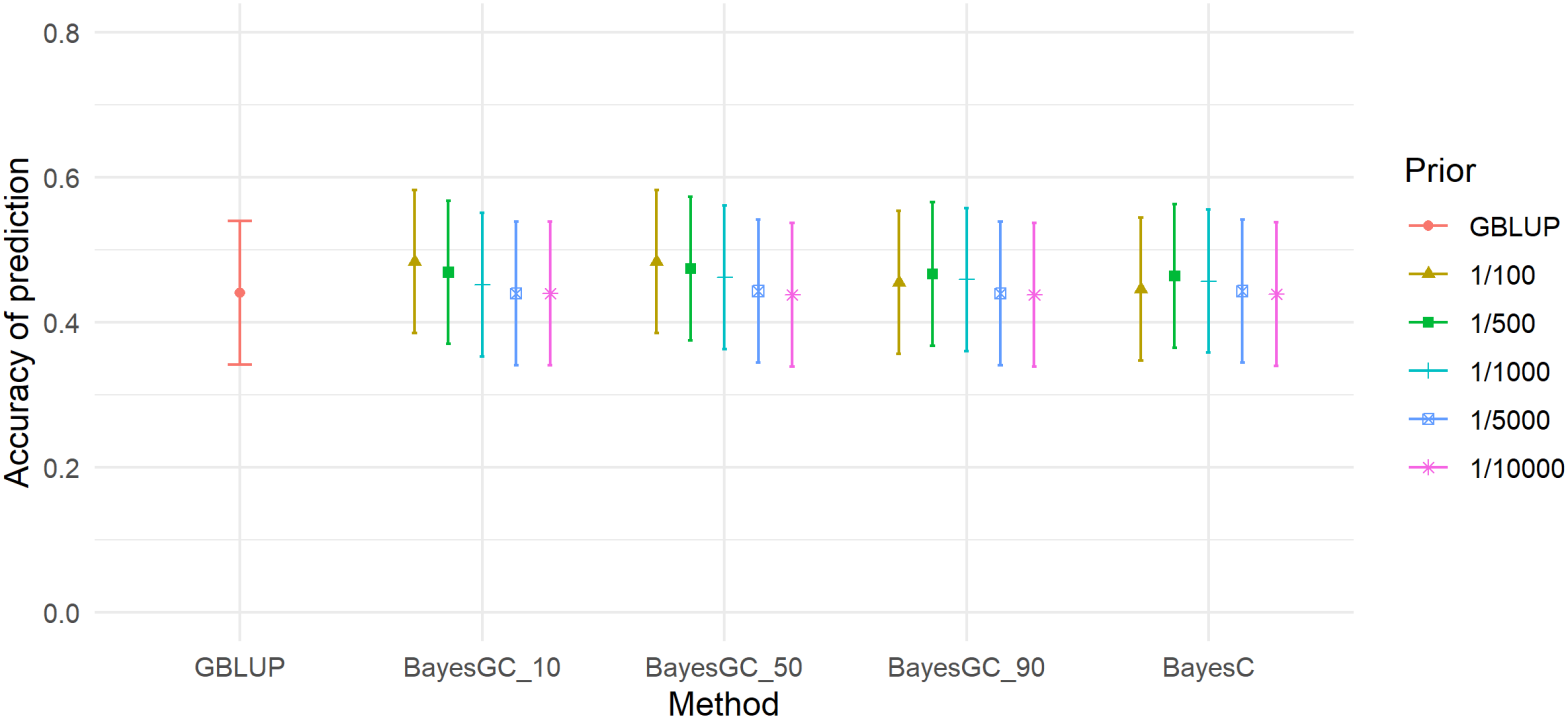
The accuracy of prediction for the trait mortality within 3 weeks (M3W), from the different prediction methods at the different priors for fraction of variance explained by a single SNP (bars denote standard errors) [Colour figure can be viewed at wileyonlinelibrary.com]

**FIGURE 4:**
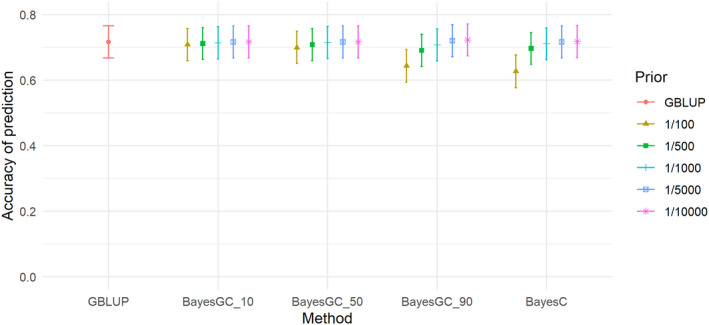
The accuracy of prediction for the trait total litter weight at 3 weeks (LW3W), from the different prediction methods at the different priors for fraction of variance explained by a single SNP (bars denote standard errors) [Colour figure can be viewed at wileyonlinelibrary.com]

For STB, there were also minor, but no significant differences in accuracy between the methods, with the highest accuracy achieved by BayesGC_10 (Fr 1/5000) and GBLUP at 0.318 and the lowest accuracy for STB was BayesC (Fr 1/100) at 0.272 (see Figure 2). M3W (Figure 3) is the trait with the largest differences between the methods. GBLUP and BayesC (Fr 1/500) had an accuracy of 0.441 and 0.464, respectively, while the highest accuracy from the BayesGC methods was achieved by BayesGC_50 (Fr 1/100) with an accuracy of 0.484 (Table 6), making a difference of 9.8% between GBLUP and BayesGC. However, the difference was not significant. For the trait LW3W (Figure 4) all the methods had high accuracies of 0.717, 0.722 and 0.718 for the methods GBLUP, BayesGC_90 (Fr 1/10,000) and BayesC (Fr 1/10,000), respectively.

**FIGURE 5:**
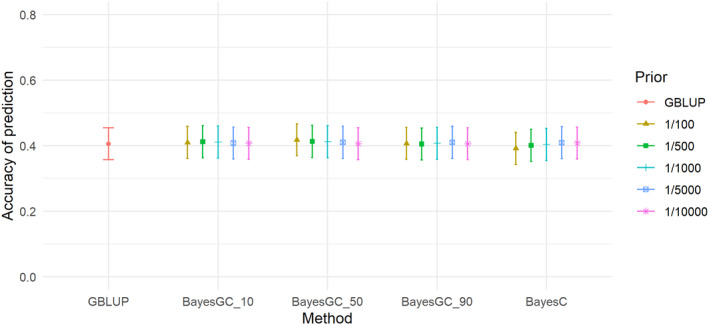
The accuracy of prediction for the trait shoulder lesions (SHL), from the different prediction methods at the different priors for fraction of variance explained by a single SNP (bars denote standard errors) [Colour figure can be viewed at wileyonlinelibrary.com]

**FIGURE 6:**
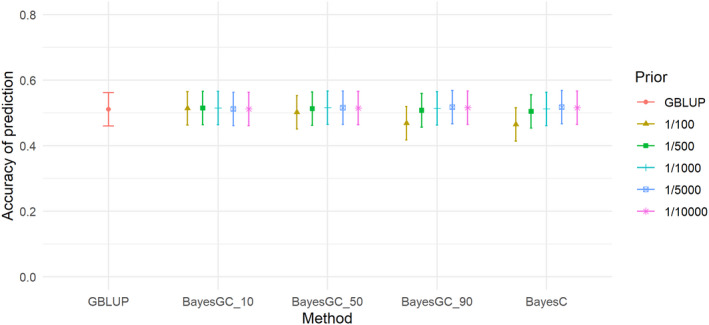
The accuracy of prediction for the trait body condition score (BCS), from the different prediction methods at the different priors for fraction of variance explained by a single SNP (bars denote standard errors) [Colour figure can be viewed at wileyonlinelibrary.com]

Shoulder Lesions (SHL) (Figure 5) showed a prediction accuracy of 0.406 and 0.409 for GBLUP and BayesC while the highest accuracy for BayesGC was 0.418 for BayesGC_50 (Fr1/100). Trait BCS (Figure 6) also had minor differences between the methods and obtained the highest accuracy from the BayesC and BayesGC_90 (Fr 1/10,000) methods with an accuracy of 0.518 for both methods, while GBLUP obtained an accuracy of 0.511.

The regression coefficients for the method yielding the highest accuracy were also the regression coefficient closest to 1 for all the traits except L3W3. L3W3 was the only trait with a regression coefficient above 1, indicating the variance of the GEBV being slightly lower compared to the yield deviations. TNB was the trait with a regression coefficient closest to 1 with a regression coefficient of 0.966 while SHL was the trait with a regression coefficient furthest from 1 at 0.436 (see Table 6), implying that the variance of the GEBV for SHL was inflated.

## 4 DISCUSSION

### 4.1 Genomic prediction methods

We have compared GBLUP, BayesGC and BayesC for six maternal traits with different priors for BayesC and BayesGC. In general, for all traits, one of the BayesGC methods yielded the highest prediction accuracy (Table 6), although its accuracy was often, but not always, matched by GBLUP and for one trait (BCS) by BayesC. This implies that fitting a combination of individual SNPs with large effects and a polygenic effect often yielded the highest prediction accuracy; however, the differences were not significant. The traits M3W and SHL yielded a 9.8% and 3.0% increase in accuracy when moving from GBLUP to BayesGC_50 (Fr1/100) (Table 6). The trait BCS had a somewhat increased accuracy of prediction (1.4% higher than GBLUP) when fitting either BayesC (Fr 1/5000) or BayesGC_50 (Fr 1/5000). The trait LW3W had a 0.7% higher accuracy for BayesGC_90 (Fr 1/10,000) than GBLUP. The traits TNB and STB showed no benefit of fitting Bayesian variable selection methods compared with GBLUP.

A limited increase in accuracy when going from GBLUP to BayesGC could be because the accuracy of prediction for the trait using GBLUP already is quite high. Our reference population was quite large (9–15,000 animals). A reference population of 7–11,000 animals was sufficient to obtain GEBV prediction accuracies comparable to the EBVs obtained with progeny testing for Japanese Black cattle (Takeda et al., 2021). TNB and LW3W with an accuracy of ~0.6 and ~0.7 for GBLUP, respectively, might not have as much potential for increasing their accuracy as M3W, with a much lower general accuracy of prediction (~0.44 for GBLUP). However, the trait STB showing the least benefit of fitting a Bayesian model also has the lowest general prediction accuracy of ~0.3. This could mean that there are other factors impacting the possible prediction accuracy of STB. For example, there could be fewer or no major QTL for the trait STB, lower linkage disequilibrium between markers and QTL, or low minor allele frequency of QTL for STB.

### 4.2 Genetic architecture

The accuracy of GP depends on the proportion of genetic variance captured by the markers, the size of the reference population, the additive genetic relationship between the animals in the reference and the validation population, the heritability of the trait, the number of independent QTL and the effective number of chromosome segments (Daetwyler et al., 2010, 2013; Habier et al., 2007, 2010; Wientjes et al., 2013). Most individuals have records for all traits in our current data, which implies that the genetic relationships between the reference and validation populations are approximately the same over the six traits. However, some individuals have missing records for some traits, resulting in reduced reference population size. M3W and LW3W have ~10 K reference animals, while the other traits have ~15 K reference populations. LW3W, SHL and BCS have the highest heritability, implying more informative reference data (Table 1). Thus, it seems that the main differences between the traits in our study are the genetic architectures of the traits, i.e. how much genetic variance is captured by the markers and the size and number of major QTLs present for each trait.

While the traits are all considered to be complex and polygenic, some of the traits might have major genes and SNPs in close linkage disequilibrium that explain a substantial part of the genetic variance. However, if there happen to be many SNPs with substantial LD to a major gene, e.g. due to high genetic drift in the region, the GBLUP method may still perform well, since it can use many SNPs to explain the major gene effect. Also, for some traits, genomic predictions may have been over larger genetic distances, i.e. reduced relationships between reference and validation animals, which favours variable selection genomic prediction methods since they focus on SNPs that are in close LD with the QTL (Meuwissen & Goddard,  2010; Solberg et al., 2009).

The QTL database (Pig QTLDdb; Hu et al., 2022) for each trait shows that there were 228 detected QTL for “Total number born alive” (TNB) and 138 QTL for “Number of stillborn” (STB). The trait “Piglet mortality within 3 weeks” (M3W) did not exist in the database. However, 10 QTL were found for the trait “Piglet Mortality”. There was also no trait in the database defined as “Total litter weight within 3 weeks” (LW3W), but 1 QTL was listed for “Total litter weight at weaning” (He et al., 2021). There was also no QTL listed for Body Condition Score (BCS) or Shoulder Lesions (SHL). However, the published QTL listed in the database does not only reflect the genetic architecture of the traits but serve also as indicators for which traits that are more or less investigated.

QTL markers identified by GWAS on sequence data may be included in 50 k marker panels for genomic prediction. In Holstein cattle (Brøndum et al., 2015), this method showed increased reliability of genomic prediction, especially when the QTL is included as a separate variance component, as it allows for extra emphasis on the QTL.

If large QTL included in the prediction model can help increase the prediction accuracy, why not just include the QTL directly in the linear model? This, however, requires a two‐step approach, where one first finds the QTL associated with the trait and then includes them in the genomic prediction model. The BayesGC method fits both the polygenic trait and the important SNPs in one analysis. Both approaches do, however, show that there is room for improvement in prediction accuracy by including important SNPs with higher emphasis in a genomic prediction model. Bayesian variable selection methods also have the potential to find the functional SNPs to include in a linear model (Meuwissen et al., 2021; van den Berg et al., 2013).

### 4.3 Prior distributions

Bayesian variable selection methods use priors, which need to be carefully chosen or hyper‐parameters of the priors estimated as part of the prediction method. The latter would extend the number of MCMC cycles substantially, as these hyper‐parameters converge much slower to their equilibrium distribution than GEBVs. In our study we tried a range of different priors, varying both the number of SNPs to be included in the model through Fr, the emphasis of each SNP through the variance explained by markers ($\sigma_{m}^{2}$) and the ratio between variance explained by markers and variance explained by the polygenic effect ($\sigma_{\mathrm{pol}}^{2}$), where with BayesGC_10, 10% of the total genetic variance is fitted with markers ($\sigma_{m}^{2}$) and 90% with the polygenic effect ($\sigma_{\mathrm{pol}}^{2}$), BayesGC_50 the variance is split 50/50 and with BayesGC_90, 90% of the total genetic variance is fitted with markers and 10% with the polygenic effect. For the Bayesian methods, the priors on the fraction of variance explained by a single SNP (Fr) seem more important than how much genetic variance is explained by either polygenic effect ($\sigma_{\mathrm{pol}}^{2}$) or the marker effects ($\sigma_{m}^{2}$), i.e. there are more differences within the methods BayesGC_10, BayesGC_50 or BayesGC_90 than between them.

M3W (Figure 3) showed the largest differences in accuracy between GBLUP and BayesGC and it seems the accuracy increases gradually as Fr becomes larger (fewer SNPs fitted) but only when the ratio between $\sigma_{m}^{2}$ and $\sigma_{\mathrm{pol}}^{2}$ is favouring $\sigma_{\mathrm{pol}}^{2}$ in such a way that the model fits 50%–90% of the genetic variance as $\sigma_{\mathrm{pol}}^{2}$ and the remaining variance is fitted with very few SNPs that in turn get fitted with a relatively high emphasis through Fr. The traits SHL and BCS (Figures 5 and 6) show a similar pattern, i.e. fitting a few SNPs is not improving the overall prediction accuracy unless it is also accompanied by a high emphasis on $\sigma_{\mathrm{pol}}^{2}$. This could indicate that finding QTL and fitting them on their own is not sufficient to obtain high prediction accuracy. One also needs the support of a polygenic effect through, e.g. a genomic relationship matrix. However, when fitting many SNPs through the BayesC term, the Bayesian variable selection method would also fit many SNPs with a small effect—similar to GBLUP. The benefit of a Bayesian variable selection method compared with GBLUP is thus expected to be lower for the methods with a higher π‐value, like the Fr 1/5000 and 1/10,000.

### 4.4 Further developments

Further development of the BayesGC would be to expand the model to include nongenotyped animals in the estimation of breeding values through, e.g. single‐step genomic prediction (Christensen & Lund, 2010; Fernando et al., 2014; Legarra et al., 2009). The challenge of including nongenotyped animals with genomic prediction is the need to impute genotypes. With linear methods, there are methods such as ssGBLUP (Aguilar et al., 2010; Christensen & Lund, 2010; Legarra et al., 2009; Misztal et al., 2009) where an additive relationship matrix H is combining information from both pedigree and SNP data. Bayesian methods for combining genotyped and nongenotyped animals have been developed that could be adapted to this model by imputing genotypes for nongenotyped animals using MCMC methods that could be used with whole‐genome data (Fernando et al., 2014). BayesGC includes a polygenic effect in the form of a **G**‐matrix, which could also be exchanged with an **H**‐matrix to include nongenotyped animals, and the marker‐model‐based single‐step approach of Fernando et al. (2014) could be used for the additional SNPs fitted by the BayesGC model. Other options for using BayesGC results in routine genomic evaluations would be to use the analysis of genotyped animals to find SNPs that need extra weight. In a regular GBLUP/ssGBLUP analysis, these SNPs would thus attain extra weights when constructing **G** and implicitly the **H**‐matrix. The information on SNP variance from a Bayesian analysis could thus be used to improve the genomic relationship matrix for GBLUP or ssGBLUP analyses.

Another way to improve BayesGC could be to expand the software towards multi‐trait analyses as many routine breeding evaluations today are based on multi‐trait models. Expanding the BayesGC model towards multi‐trait analyses is relatively straightforward if one assumes that an SNP with a large effect, is affecting all the (related) traits (Karaman et al., 2019; Kemper et al., 2018). In situations where one cannot assume this, multi‐trait variable selection modelling requires to sample which combination of traits is affected by each of the SNPs. If there are many traits, there are many such combinations. Applying the BayesGC results to multi‐trait routine evaluations may be by giving extra weight to some SNP genotypes, resulting in a different **G**‐matrix for each of the traits, and consequently also for different pairs of traits (since the **G**‐matrix modelling covariances between traits i and j is constructed as the cross‐product of the SNP genotypes weighted for trait i and those weighted for trait j). Modifications of routine software packages may be needed to accommodate these per trait alternative **G**‐matrices.

Bayesian variable selection methods have a lot of potential for further development to be used in routine breeding value estimations. One of the biggest drawbacks today is the high computational costs of running the MCMC chains. However, computational power has historically increased and will most likely continue to increase, in addition to further research developing into more efficient algorithms using parallel computations.

## 5 CONCLUSIONS

The accuracy of genomic prediction on six maternal traits in landrace pigs varied greatly ranging from 0.31 to 0.61. The prediction accuracies did not vary much between the different genomic prediction methods. The two traits M3W and BCS could benefit from using a BayesGC approach with a 9.8 and 3.0% increase in accuracy, respectively, while TNB, STB, LW3W and SHL showed only minor improvements. Although GBLUP, BayesC and BayesGC all yielded similar genomic prediction accuracies, the accuracy of BayesGC was always as high as or higher than that of GBLUP. Within the BayesGC method, the accuracies could vary depending on the prior distributions. The models were more sensitive to how many markers were fitted in the model by varying the fraction of the total genetic variance explained by a single marker (Fr) compared with the amount of total genetic variance explained by marker effects as a whole (BayesGC_10, BayesGC_50 or BayesGC_90), but overall, most traits were robust against varying the prior distributions.

## CONFLICT OF INTEREST

The authors confirm that there is no known conflict of interest associated with this publication.

## Data Availability

Restrictions apply to the availability of these data, which were used under licensed for this study. Data might be available upon reasonable request from the authors with the permission of Topigs Norsvin.
